# Activation of Wnt/β-catenin signalling *via* GSK3 inhibitors direct differentiation of human adipose stem cells into functional hepatocytes

**DOI:** 10.1038/srep40716

**Published:** 2017-01-17

**Authors:** Jieqiong Huang, Xinyue Guo, Weihong Li, Haiyan Zhang

**Affiliations:** 1 Department of Cell Biology, Municipal Laboratory for Liver Protection and Regulation of Regeneration, Capital Medical University, Beijing, China

## Abstract

The generation of hepatocytes that are derived from human adipose stem cells (hASCs) represents an alternative to human hepatocytes for individualized therapeutic and pharmaceutical applications. However, the mechanisms facilitating hepatocyte differentiation from hASCs are not well understood. Here, we show that upon exposure to glycogen synthase kinase 3 (GSK3) inhibitors alone, the expression of definitive endoderm specific genes *GATA4, FOXA2,* and *SOX17* in hASCs significantly increased in a manner with activation of Wnt/β-catenin signalling. Down regulation of the β-catenin expression attenuates the effect of GSK3 inhibitors on the induction of these specific genes. The cells induced using GSK3 inhibitors were directed to differentiate synchronously into hepatocyte-like cells (HLCs) after further combinations of soluble factors by a reproducible three-stage method. Moreover, hASC-HLCs induced using GSK3 inhibitors possess low-density lipoprotein uptake, albumin secretion, and glycogen synthesis ability, express important drug-metabolizing cytochrome P450 (CYP450) enzymes, and demonstrate CYP450 activity. Therefore, our findings suggest that activation of Wnt/β-catenin signalling *via* GSK3 inhibitors in definitive endoderm specification may represent an important mechanism mediating hASCs differentiated to functional hepatocyte. Furthermore, development of similar compounds may be useful for robust, potentially scalable and cost-effective generation of functional hepatocytes for drug screening and predictive toxicology platforms.

The utilization of human primary hepatocytes for both therapeutic and pharmaceutical purposes is limited by shortage of donors, batch variation in hepatic functionality and dedifferentiating with time in culture^1^. Therefore, alternative sources of human hepatocytes are urgently required. Recent studies have demonstrated that hepatocytes derived from human adipose stem cells (hASCs) are potentially scalable and applicable alternative to human hepatocytes^2^,^3^,^4^,^5^. However, the signalling mechanisms facilitating hepatocyte differentiation from hASCs are not well understood. In the liver development, definitive endoderm specification is the essential early and the most important step to generate of hepatocytes. Thus, a better understanding and control of the definitive endoderm differentiation process *in vitro* should result in enhanced efficiency and higher fidelity in the resulting cells^6^,^7^. The efficient and reproducible production of definitive endoderm is dependent on our ability to recapitulate key stages of embryonic lineage development in differentiation cultures. During gastrulation and patterning of endoderm in mammalian, TGFβ/Nodal and Wnt signalling result in an anterior region with potential to form the definitive endoderm from which the hepatic endoderm is generated. Nodal signalling stimulates the expression of a core group of endoderm transcription factors including the HMG domain DNA-binding factor SOX17 and the fork head domain proteins FOXA1–3 which in turn regulate a cascade of genes committing cells to the endoderm lineage^8^. Wnt signalling combined with fibroblast growth factor (FGF) and bone morphogenetic protein (BMP) signalling regulates foregut endoderm identity dependent on the graded activity of Wnt. A secreted frizzled-related protein 5, Wnt ligand and frizzled (Fzd) 7 interactions regulate differential thresholds of Wnt/β-catenin and Wnt/JNK signalling that coordinate endoderm fate, proliferation and morphogenesis^6^,^9^. Previously, we demonstrate that the high concentration (100 ng/mL) of activin A signalling, which mimics the Nodal pathway, induces definitive endoderm specific transcription factors, including HEX, GATA4, FOXA2, and SOX17, expression in hASCs^10^. But the effect of Wnt signalling during this process is still unclear. Recent studies suggest that Wnt signalling is required to specify definitive endoderm from human embryonic stem cells (hESCs) and human induced pluripotent stem cells (hiPSCs). Manipulations of Wnt signalling *via* glycogen synthase kinase 3 (GSK3) inhibitors have been exploited to direct differentiation of definitive endoderm and hepatocyte^11^,^12^,^13^,^14^,^15^,^16^,^17^. However, whether Wnt signalling or inhibiting GSK3 can be used for determining definitive endoderm fate and for generation of hepatocytes from hASCs is not clear.

GSK3 is a serine/threonine kinase that plays a central role in the regulation of the Wnt/β-catenin signalling pathway, an important pathway for hepatic specification, hepatoblast proliferation, differentiation, and hepatocyte maturation^18^,^19^,^20^. When the Wnt ligand is present, it binds its receptor Fzd and the coreceptor lipoprotein-related protein 5 and 6 (LRP-5/6) on the target cell, which signals through dishevelled (Dvl) to suppress β-catenin phosphorylation; β-catenin is able to complex with T-cell factor/lymphoid enhancer-binding factor (TCF/LEF) and induce target gene transcription^21^. In the resting state, GSK3 and casein kinase I (CKI) phosphorylate β-catenin, triggering its destabilization and degradation to maintain a very low level of β-catenin in the cytosol/nucleus. Thus, pharmacologic inhibition of GSK3 activity can lead to stabilization and activation of β-catenin and TCF/LEF-dependent gene transcription, which reflects the activity of Wnt signal transduction^22^. Recent studies suggest that downstream of GSK3 inhibition, elevated cMyc and β-catenin act in parallel to reduce transcription and DNA binding, respectively, of the transcriptional repressor Tcf7l1. Tcf7l1 represses FOXA2, a pioneer factor for endoderm specification^23^.

Because small molecules provide a highly temporal and tunable approach to modulate cellular fate and functions, they have been identified to enhance and enable stem cell differentiation towards a faster, more efficient, and directed process^24^. In this study, we compared the effect of Wnt3a and two GSK3 inhibitors, Chir98014 and Chir99021 on the activation of Wnt/β-catenin signalling pathway, and the regulation of expression of definitive endoderm specific transcription factors GATA4, FOXA2, and SOX17 in hASCs. Thereafter, we investigated whether the cells inducing by GSK3 inhibitors may show equivalent developmental potential as activin A-induced definitive endoderm in their differentiation into functional hepatocytes from hASCs *in vitro*. This study may provide a useful approach for more cost-effective hepatocyte production for drug discovery and ultimately cell therapy.

## Results

### GSK3 inhibitors activated Wnt/β-catenin signalling axis in hASCs

**Figure 1:**
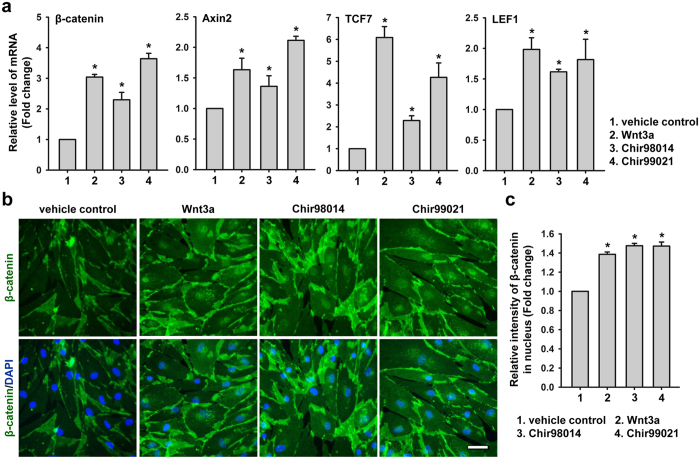
GSK3 inhibitors activated Wnt/β-catenin signalling axis in hASCs. (**a**) Gene expression in hASCs after treatment for 24 hours with different factors was determined. *Statistical significance compared to control, *p* < 0.05. (**b**) Immunofluorescence staining for β-catenin in hASCs after treatment for 24 hours with different factors. Bars, 100 μm. (**c**) Expression of β-catenin in the nuclei of hASCs after treatment for 24 hours with different factors was determined using HCS platform. *Statistical significance compared to control, *p* < 0.05.

To examine whether inhibition of GSK3 would mimic Wnt signalling through direct stabilization of β-catenin in hASCs, the cells after 48 hours of serum starvation were treated with 50 ng/mL Wnt3a, 0.2 μM Chir98014 and 2 μM Chir99021 for 24 hours separately. The mRNA levels of key factors in Wnt signalling, including β-catenin, Axin2, TCF7, and LEF1, in hASCs were quantified by real-time RT-PCR. The results showed that the mRNA levels of these genes were significantly increased compared to the vehicle control (Fig. 1a). Meanwhile, we found that the protein levels in nucleus of β-catenin in Wnt3a-treated cells, Chir98014-treated cells, and Chir99021-treated cells were significantly higher than the vehicle control cells (Fig. 1b,c). However, there were no difference between the Wnt3a-treated cells and GSK3 inhibitors-treated cells (Fig. 1c). These results suggest that the canonical Wnt/β-catenin axis was activated in Wnt3a, Chir98014 and Chir99021 treated hASCs.

### GSK3 inhibitors up-regulate the expression of definitive endoderm specific genes dependent on Wnt/β-catenin signalling

**Figure 2:**
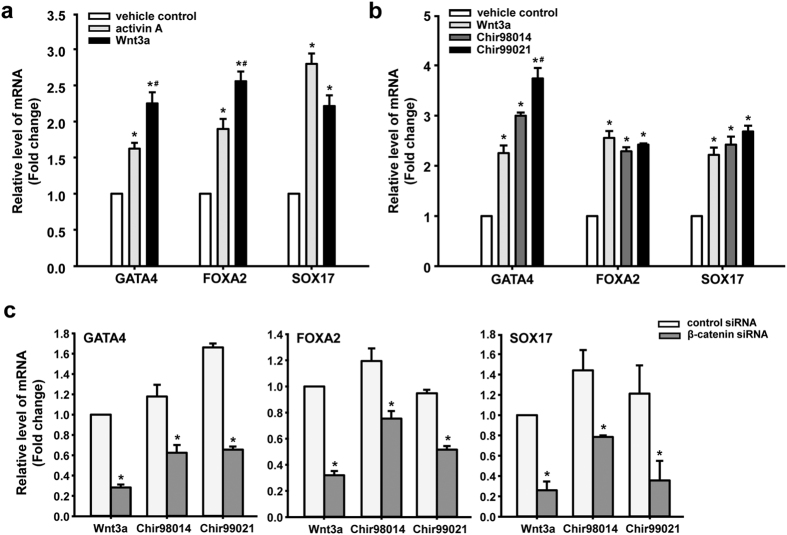
GSK3 inhibitors regulated definitive endoderm specification of hASCs *via* Wnt/β-catenin signalling. Gene expression in hASCs after treatment for 24 hours with different factor was analysed (**a**) and (**b**). *Statistical significance compared to control, *p* < 0.05; ^#^Statistical significance compared to activin A (**a**) or compared to Wnt3a (**b**), *p* < 0.05. (**c**) Gene expression in hASCs post-siRNA transfection, and then treated with different factors for 24 hours. *Statistical significance compared to control siRNA, *p* < 0.05.

To investigate whether the activation of Wnt signalling by Wnt3a and GSK3 inhibitors affected definitive endoderm specification from hASCs, we compared firstly the effect of Wnt3a and activin A on the regulation of the definitive endoderm specific genes *GATA4, FOXA2,* and *SOX17*. The results showed that 24 hours of exposure, the expression levels of these definitive endoderm markers in Wnt3a-treated cells and in activin A-treated cells were significantly increased compared to the vehicle control (Fig. 2a). Furthermore, the mRNA levels of GATA4 and FOXA2 in Wnt3a-treated cells was significantly higher than in activin A-treated cells (Fig. 2a). Then, we examined whether the GSK3 inhibitors could up-regulate the expression of definitive endoderm specific genes in hASCs. We treated hASCs with 0.2 μM Chir98014 and 2 μM Chir99021 for 24 hours. Wnt3a was used as a positively control. Consistent with changes in Wnt3a-treated cells, a pulse exposure of Chir98014 and Chir99021 can significantly increase the expression levels of GATA4, FOXA2, and SOX17 compared to the vehicle control (Fig. 2b). Likewise, the mRNA levels of GATA4 in Chir99021-treated cells were significantly higher than in Wnt3a-treated cells (Fig. 2b). Time course analyses indicated that GSK3 inhibitors-based conditions induced a peak expression of these definitive endoderm markers on day 1 (Supplemental Fig. S1). The expression of the definitive endoderm markers in hASCs did not increase with longer exposure to GSK3 inhibitors.

To assess whether the effect of GSK3 inhibitors on induction definitive endoderm specification depends on the β-catenin signalling pathway, we disrupted the signalling pathway by delivering a siRNA to knock down the expression of *β-catenin* gene. As shown in Supplemental Figure S2, β-catenin expression was successfully reduced by 50% compared to the control siRNA group at the mRNA levels (Supplemental Fig. S2a). Immunofluorescence staining and high content screening (HCS) quantitative analyses showed that the protein level of β-catenin expression in β-catenin siRNA group was significantly lower than the cells in control siRNA group (Supplemental Fig. S2b,c).

By reducing the β-catenin expression level using siRNA, the effects of Wnt3a, Chir98014 and Chir99021 on definitive endoderm specification were assessed. The results showed that the reduction of β-catenin did not impact the expression of definitive endoderm specific genes (Supplemental Fig. S3), but it significantly decreased the mRNA levels of GATA4, FOXA2, and SOX17 in cells following treatment with Wnt3a or GSK3 inhibitors for 24 hours (Fig. 2c). These data showed that GSK3 inhibitors induce definitive endoderm specification of hASCs dependent on the activity of canonical Wnt/β-catenin signalling.

### Production of definitive endoderm cells using GSK3 inhibitors

**Figure 3:**
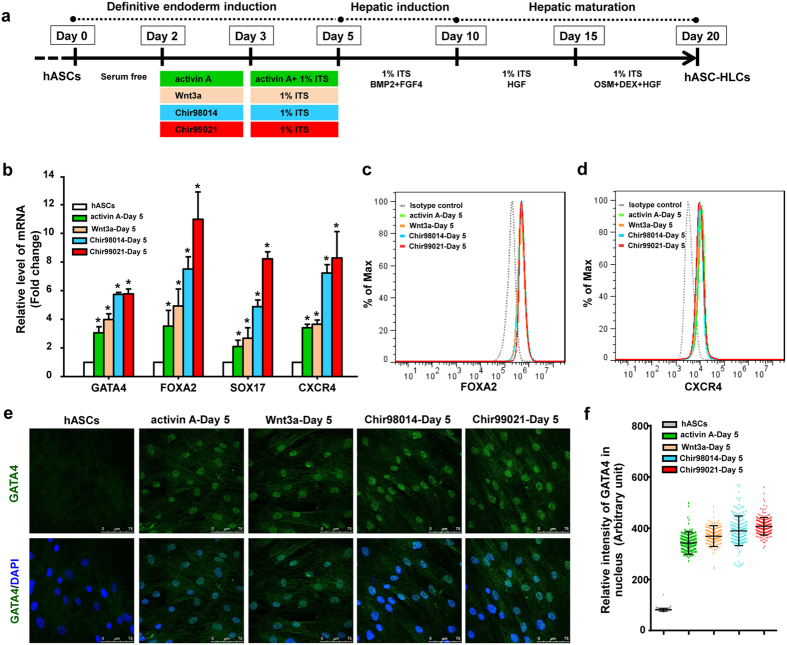
Properties of differentiated cells at the definitive endoderm differentiation phase. (**a**) Protocol for the definitive endoderm induction, hepatic induction, and hepatic maturation. (**b**) Gene expression in hASCs and differentiated cells at day 5 was analysed. *Statistical significance compared to hASCs, *p* < 0.05. Flow cytometric analysis of FOXA2 (**c**) and CXCR4 (**d**) expression. (**e**) Immunofluorescence staining for GATA4 in hASCs and differentiated cells. Bars, 75 μm. (**f**) Expression of GATA4 in the nuclei of hASCs and differentiated cells was determined using HCS platform.

To determine the production of definitive endoderm cells using GSK3 inhibitors, the cells were differentiated using a three-stage differentiation protocol as previously with modification^10^. During definitive endoderm induction, hASCs were divided into four groups (Fig. 3a). Briefly, at day 2, hASCs were incubated separately with 100 ng/mL activin A for 72 hours, 50 ng/mL Wnt3a, or 0.2 μM Chir98014, or 2 μM Chir99021 for 24 hours, 1% insulin-transferrin selenium (ITS) was added to the medium beginning at day 3. At day 5, the properties of differentiated cells in four groups were analysed. The results showed that the mRNA levels of definitive endoderm cells markers, including GATA4, FOXA2, SOX17 and C-X-C motif chemokine receptor 4 (CXCR4) were significantly increased compared to hASCs (Fig. 3b). To verify the differentiation efficiency, the expression of FOXA2 and CXCR4 in cells was examined by flow cytometric analysis. The data showed that the percentage of FOXA2 positive cells in four treated group was 78.8% (activin A-treated cells), 71.5% (Wnt3a-treated cells), 77.1% (Chir98014-treated cells), and 72.2% (Chir99021-treated cells) (Fig. 3c), and the percentage of CXCR4 positive cells in four treated group was 72.1% (activin A-treated cells), 73.1% (Wnt3a-treated cells), 65.4% (Chir98014-treated cells), and 62.3% (Chir99021-treated cells) separately (Fig. 3d). Immunofluorescence staining showed that the cells in four groups exhibited similar nucleus patterns of expression of GATA4 (Fig. 3e). HCS quantitative analyses for the expression of GATA4 demonstrated that the level of GATA4 proteins in response to Chir98014 and Chir99021 treatment with a comparable efficiency as that of activin A treatment, which were significantly higher than the hASCs (Fig. 3f). These findings demonstrate that Chir98014 and Chir99021 treatment may switch hASCs developmental fate and committed to the definitive endoderm lineage.

### Definitive endoderm cells induced by GSK3 inhibitors possess the potential to differentiate into hepatocyte-like cells

**Figure 4:**
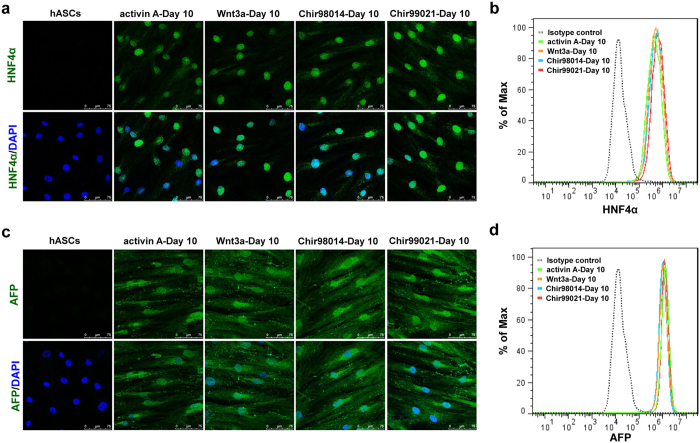
Properties of differentiated cells at the hepatic specification phase. Immunofluorescence staining for HNF4α (**a**) and AFP (**c**) in hASCs and differentiated cells. Bars, 75 μm. Flow cytometric analysis of HNF4α (**b**) and AFP (**d**) expression in hASCs and differentiated cells at day 10.

The properties of differentiated cells in four groups were analysed at day 10 and day 20. Immunofluorescence staining data verified that the cells in the four groups were positive for hepatocyte nuclear factor 4 alpha (HNF4α) (Fig. 4a) and α-fetoprotein (AFP) (Fig. 4c), which are markers of hepatic progenitor cells. To verify the differentiation efficiency, the expression of HNF4α and AFP in cells at day 10 was examined by flow cytometric analysis. The data showed that the percentage of HNF4α positive cells in four treated group was 86.9% (activin A-treated cells), 91.8% (Wnt3a-treated cells), 92.5% (Chir98014-treated cells), and 96% (Chir99021-treated cells) (Fig. 4b), and the percentage of AFP positive cells in four treated group was 99.3% (activin A-treated cells), 98.9% (Wnt3a-treated cells), 98.8% (Chir98014-treated cells), and 99.2% (Chir99021-treated cells) separately (Fig. 4d). These results indicated that almost all of the cells induced by GSK3 inhibitors synchronously acquired the properties of hepatic progenitor cells following 5 days of hepatic induction.

**Figure 5:**
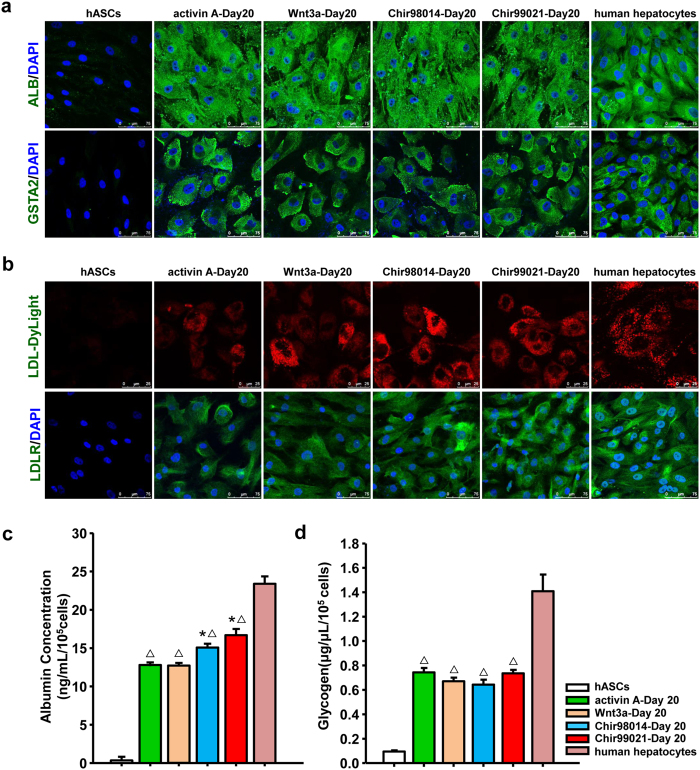
Characterization of hASC-HLCs. (**a**) Immunofluorescence staining for ALB and GSTA2 in hASCs, differentiated cells at day 20, and human hepatocytes. Bars, 75 μm. (**b**) LDL-DyLight uptake and immunofluorescence staining for LDLR in hASCs, differentiated cells at day 20, and human hepatocytes, Bars, 25 μm and 75 μm. (**c**) Albumin secretion was analysed in hASCs, differentiated cells and human hepatocytes. (**d**) Glycogen production was assessed in hASCs, differentiated cells, and human hepatocytes. *Statistical significance compared to hASC-HLCs-activin A, *p* < 0.05; ^Δ^Statistical significance compared to human hepatocytes, *p* < 0.05.

Following a 10-day maturation process, the morphology of the cells became the polygonal epithelial cells-like. Quantitative comparisons of gene expression revealed that the mRNA levels of hepatic functional markers, including apolipoprotein C1 (APOC1), phosphoenolpyruvate carboxykinase 2 (PCK2), and carbamoyl-phosphate synthase 1 (CPS1), in cells at day 20 were highly increased compared with the expression in hASCs. The expression levels of APOC1 in Chir98014-treated cells and Chir99021-treated cells at day 20 were relative higher than the levels in activin A-treated cells. The expression level of CPS1 in Chir98014-treated cells at day 20 was also higher than the levels in activin A-treated cells. Nevertheless, the expression levels of these genes in cells at day 20 were relative lower than the levels in human hepatocyte (Supplemental Fig. S4). Immunofluorescence staining analysis showed that at day 20 all of the cells in four groups expressed albumin (ALB) and glutathione S-transferase alpha 2 (GSTA2), which were similar with human hepatocytes (Fig. 5a). Meanwhile, the expression of AFP, marker of hepatic progenitor cells, was dramatically decreased in cells at day 20 with different factors (Supplemental Fig. S5).

Low-density lipoprotein (LDL) uptake examination showed that the internalized LDL-DyLight^TM^ 550 particles presented in the cells of four groups. The percentage of cells with LDL particles in cells of Chir99021 at day 20 was relatively higher than other groups, which resembles the human hepatocytes (Fig. 5b). Immunofluorescence staining analysis showed that at day 20 all of the cells in four groups expressed LDL receptor (LDLR) as well as human hepatocyte (Fig. 5b). ALB secretion determination showed that the cells derived from Chir98014 and Chir99021 groups secreted ALB at a level of 11.2 ± 0.02 ng/mL and 14.8 ± 0.02 ng/mL per 10^5^ cells, which was greater than the level of in cells derived from activin A or Wnt3a (Fig. 5c). The level of secreted ALB in the cells derived from Chir98014 and Chir99021 was still lower than the level of in human hepatocytes (Fig. 5c). Glycogen synthesization determination showed that the level of glycogen in cells derived from four groups significantly higher than the levels of in hASCs (Fig. 5d). The level of glycogen in the cells from different groups was also lower that the level of in human hepatocytes (Fig. 5d).

These data indicate that hASCs induced by Wnt3a, Chir98014 and Chir99021 at stage of endoderm induction can be differentiated into hepatocyte-like cells (HLCs) that exhibit mature hepatocyte-specific protein and function following the favour hepatic differentiation and maturation induction. Therefore, we named HLCs from four groups as hASC-HLCs-activin A, hASC-HLCs-Wnt3a, hASC-HLCs-Chir98014, and hASC-HLCs-Chir99021.

### hASC-HLCs express cytochrome P450 (CYP450) enzymes and have CYP450 activity

To further evaluate the expression and activity of CYP450 in hASC-HLCs induced using GSK3 inhibitors, quantitative comparisons of the mRNA levels of the CYP450 subfamily was examined firstly (Supplemental Fig. S6). The expression of CYP1A2 in hASC-HLCs-Wnt3a, hASC-HLCs-Chir98014, and hASC-HLCs-Chir99021 was significantly higher than the expression in hASC-HLCs-Activin A. The expression of CYP7A1 in hASC-HLCs-Wnt3a, and hASC-HLCs-Chir99021 was significantly higher than the expression in hASC-HLCs-Activin A. The expression of CYP1A2, CYP2B6, CYP3A4 and CYP7A1 in hASC-HLCs-Chir99021 was significantly higher than the expression in hASC-HLCs-activin A. The expression of CYP2B6, CYP3A4 and CYP7A1 in hASC-HLCs-Chir99021 was significantly higher than the expression in human hepatocytes. The expression of CYP2E1 in hASC-HLCs from four different groups was significantly higher than the expression in human hepatocyte.

**Figure 6:**
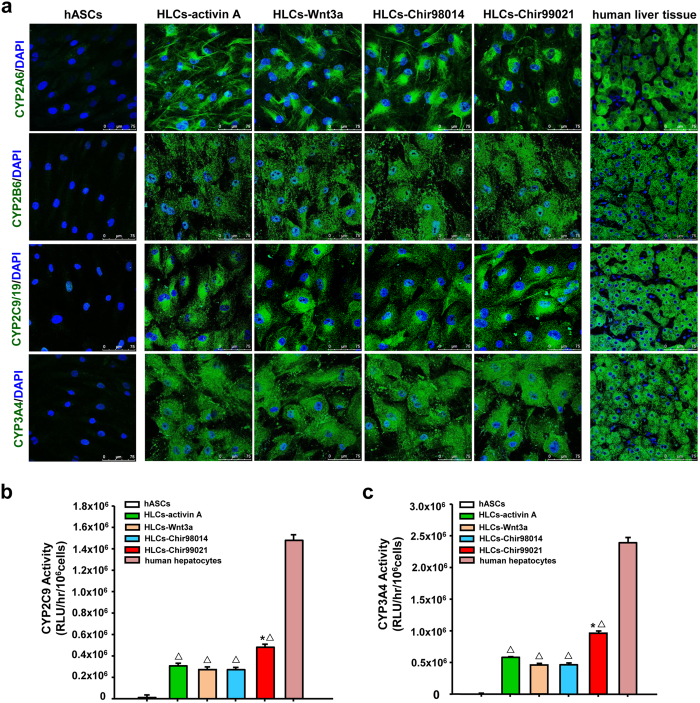
Expression and activity of CYP450 in hASC-HLCs. (**a**) Immunofluorescence staining for CYP450 in hASCs, hASC-HLCs, and human liver tissue. Bars, 75 μm. The activity of CYP2C9 (**b**) and CYP3A4 (**c**) was assessed in hASCs, hASC-HLCs, and human hepatocytes. RLU, relative luminescent unit. *Statistical significance compared to hASC-HLCs-activin A, *p* < 0.05. ^Δ^Statistical significance compared to human hepatocytes, *p* < 0.05.

Immunofluorescence analysis verified that hASC-HLCs from the four groups were positive for CYP2A6, CYP2B6, CYP2C9/19, and CYP3A4, which was similar to hepatocytes from human liver tissue (Fig. 6a).

To further demonstrate the changes in cellular function, the activities of CYP2C9 and CYP3A4 were evaluated respectively. The activity of CYP2C9 (Fig. 6b) and CYP3A4 (Fig. 6c) in hASC-HLCs-99021 was relatively higher than the hASC-HLCs-activin A, hASC-HLCs-Wnt3a, and hASC-HLCs-98014, but lower than human hepatocytes.

## Discussion

In the present study, we elucidated that pharmacological inhibition of GSK3 with the specific inhibitors Chir98014 or Chir99021 up-regulated the expression of definitive endoderm specific genes *via* activation of the Wnt/β-catenin signaling axis. hASCs from four different donors induced by GSK3 inhibitors possess the potential to differentiate into hepatocyte-like cells with mature function, including LDL uptake, ALB secretion and glycogen synthesis. Moreover, the hASC-HLCs-Chir98014 and hASC-HLCs-Chir99021 expressed the important drug-metabolizing CYP450 enzymes including CYP2A6, CYP2B6, CYP2C9/19, and CYP3A4 and the hASC-HLCs-Chir99021 had relatively higher CYP2C9 and CYP3A4 activity than other groups of hASC-HLCs. Our results demonstrate development of similar compounds may be useful for robust, potentially scalable and cost-effective generation of large numbers of functional hepatocytes for drug screening and predictive toxicology platforms.

The induction and differentiation of definitive endoderm is an essential early step to generate of hepatocytes from human pluripotent stem cells (hPSCs)^25^. Higher efficient and reproducible definitive endoderm induction will contribute to the development of homogeneous and higher fidelity end-stage populations, such as hepatocytes. Evidence indicates that the Nodal and the Wnt signalling pathway plays a pivotal role in the establishment of the early primitive streak, which ultimately leads to endoderm specification, has been identified^26^. Various groups target these two pathways in order to generate definitive endoderm from hPSCs, but the signalling mechanisms facilitating definitive endoderm induction from hASCs are not well understood. Since it is simple, robust and lower cost than growth factors, the use of small molecules that modulate the generation of specific cell types from stem cells opens new pathways to optimize protocols in stem cell differentiation^27^. Rational design or screening small molecules to generate a specific cell linage differentiated from stem cells depends on our basic understanding of human development. Studies indicates that the Wnt signalling pathway plays a pivotal role in the initiation or generation of definitive endoderm from hPSCs^28^. Thus, directly inhibiting the signalling molecule GSK3, which mimics activation of the Wnt pathway, was used to induce the differentiation of hPSCs to definitive endoderm and then to the hepatic lineage^11^,^12^,^13^.

On the other hand, Wnt signalling has been identified to control the fate of mesenchymal stem cells (MSCs)^29^. However, little is known about the role of Wnt and its downstream intracellular pathways in the differentiation of definitive endoderm and hepatic progenitor cells from hASCs. Herein, we found GSK3 inhibitors increase the expression and nuclear distribution of β-catenin. GSK3 phosphorylated β-catenin, triggering its destabilization and degradation to maintain a very low level of β-catenin in the cytosol/nucleus. Accumulated nuclear levels of β-catenin in GSK3 inhibitors treated cells indicated the GSK3 activity was inhibited. Meanwhile, the levels of members of TCF/LEF transcription factors TCF7 and LEF1 in hASCs were increased upon GSK3 inhibitors stimulation. This implied that Wnt/β-catenin signalling pathway was activated in hASCs upon GSK3 inhibitors stimulation.

The data presented here demonstrate that the GSK3 inhibitors promoted the efficient up-regulation of definitive endoderm specific transcription factors GATA4, FOXA2, SOX17 and CXCR4. Down regulating of the expression β-catenin by siRNA caused the decreased expression of these definitive endoderm specific transcription factors in hASCs after treatment with Wnt3a, Chir98014, or Chir99021. The transcription factor FOXA2 is the earliest known marker of definitive endoderm. It’s up-regulation at the onset of gastrulation is considered to represent a crucial step in commitment to definitive endoderm identity^30^. GATA4 is a zinc finger transcription factor that has important functions in several mesodermal and endoderm organs, including heart, liver and pancreas^31^. Study proposed that GATA4 is an important downstream effector of GSK3 and inhibition of GSK3 at least in part by removing its negative constraint upon GATA4^32^. In this study, Chir98014 and Chir99021 significantly increased the mRNA and protein levels of GATA4 and FOXA2 in hASCs after 24 hours exposure. At day 5, more than 75% of cells expressed the FOXA2 and CXCR4, the markers of definitive endoderm cells. The result suggested that the activity of Wnt/β-catenin signalling pathway in hASCs play a key role in definitive endoderm specification. Recent study using mouse ES cells showed that β-catenin and cMyc operate downstream of GSK3 inhibition to reduce Tcf7l1 transcription and activity, respectively. Tcf7l1 is a barrier to definitive endoderm specification, acting to directly block induction of the pioneer transcription factor, FOXA2. This explains the possible mechanism of GSK3 inhibition in promoting endoderm lineage specification^23^.

Suboptimal hepatic induction is one likely cause of the variability that can influence hepatic maturation and contribute to the development of heterogeneous end-stage populations. The efficiency of hepatic induction was monitored through changes in protein expression patterns as assessed by immunofluorescence or flow cytometry. The latter approach provides a rapid quantitative read-out but is dependent on the availability of antibodies against ideal markers of on interest on the cells. We show here that we equally successfully acquired the homogenous hepatic progenitor cells when using Chir98014 or Chir99021 induction as with activin A and Wnt3a, and then treatment with BMP2 and FGF4 for 5 days as shown previously^10^. Nearly all cells in the four groups highly expressed the hepatic progenitor markers HNF4α and AFP. At the end of the final phase, the cells induced by Chir98014 or Chir99021 treatment expressed a number of hepatic markers at the transcriptional, and protein levels. Functional determination showed that hASC-HLCs-Chir98014 and hASC-HLCs-Chir99021 exhibited mature functions of hepatocyte, including LDL uptake, ALB protein production, and glycogen synthesis.

Detoxification of exogenous compounds is one of the liver’s major metabolic functions and is mediated by a large family of proteins, including CYP450. Thus, the expression and activity of CYP450 are key markers for the HLC maturity. A recently study shows the phenotypic and functional properties of hESC-derived HLCs better mimic fetal hepatocytes with weak expression of CYP2A6, a hallmark of adult hepatocytes^16^. Our data confirmed that CYP2A6, CYP2B6, CYP2C9/19 and CYP3A4 were expressed as expected in hASC-HLCs. The abundant CYP2A6 and CYP3A4 in hASC-HLCs-Chir98014 or hASC-HLCs-Chir99021 were similar with the adult hepatocytes in a human liver tissue section. Similarly, the levels of CYP2C9 and CYP3A4 enzyme activity in hASC-HLCs-Chir99021 were relatively higher than that in hASC-HLCs-activin A. These data imply that hASC-HLCs in this study more closely resemble human hepatocytes for pre-clinical assessment of new candidate compounds in drug development^33^. Of course many efforts are still needed for promoting hASC-HLCs further maturation under the hepatic microenvironment, until they have equal metabolic abilities compared with human adult hepatocytes.

In conclusion, we demonstrate here a single chemical entity to direct differentiation of hASCs to definitive endoderm cells and towards functional hepatocytes *via* activation of the Wnt/β-catenin pathway at early stage. The generation of hASC-HLCs using the chemically directed differentiation may provide a foundation for scalable hepatocyte production for drug screening, predictive toxicology platforms and cell therapy.

## Methods

### Human subjects

Human tissues were obtained with informed patient consent and under the approval of the Ethics Committee of Capital Medical University (Beijing, China) for this project. All experimental protocols involving human tissues were reviewed and approved by the Ethics Committee of Capital Medical University. All methods involving human cells and tissue were carried out in accordance with relevant guidelines and regulations of the Ethics Committee of Capital Medical University.

### Cell culture and human tissue

hASCs from four different donors were developed as our previously reported^10^. hASCs were maintained in DMEM/F-12 (Invitrogen, Grand Island, NY, USA) supplemented with 10% fetal bovine serum mesenchyme stem cell screened (FBS-MSCS, HyClone, Logan, UT, USA), 100 U/mL penicillin, and 100 μg/mL streptomycin. At 80% confluence, the cells were passaged using 0.05% trypsin-0.02% EDTA (Sigma-Aldrich, St. Louis, MO, USA) and plated at a density of 5000 cells/cm^2^. Cells from passages six to nine were used in this study.

Human hepatocytes (ScienCell Research Laboratories, Carlsbad, CA, USA) were cultured on collagen type I-coated 24-well plates at a density of 150,000 cells/cm^2^ with hepatocyte medium (ScienCell Research Laboratories).

Human liver tissue sections were obtained from the department of pathology of beijing chaoyang hospital, and patients were informed with consent under ethical approval.

### Hepatic differentiation

hASCs were plated at a density of 40,000 cells/cm^2^ on collagen I (Invitrogen)-coated dishes (Nunc, Roskilde, Denmark) and cultured in DMEM/F-12 (Invitrogen) supplemented with 10% FBS-MSCS (HyClone), 100 U/mL penicillin, and 100 μg/mL streptomycin at 37 °C with 5% CO_2_. Once the cells reached 90% confluence, they were washed twice with PBS and incubated with serum-free DMEM/F-12 medium for 48 hours.

To induce definitive endoderm differentiation, the cells were incubated with DMEM/F-12 containing 0.5 mg/mL albumin fraction V(BSA) (Sigma-Aldrich), 50 ng/mL Wnt3a (Peprotech, Rocky Hill, NJ,USA), or GSK3 inhibitors Chir98014 (0.2 μM, Selleckchem, Houston, Texas, USA), or Chir99021 (2 μM, Selleckchem) for 24 hours, or 100 ng/mL activin A (Peprotech) for 72 hours; 1% ITS (Sigma-Aldrich) was added to the medium beginning at the second day.

For subsequent hepatic differentiation, the medium was changed to MEM/NEAA (Invitrogen), supplemented with 0.5 mg/mL BSA, 1% ITS, 20 ng/mL BMP2 (Peprotech) and 30 ng/mL FGF4 (Peprotech) for 5 days. To allow for hepatocytes maturation, the cells were further treated with 20 ng/mL Hepatocyte Growth Factor (HGF) for 5 days, and 20 ng/mL HGF, 10 ng/mL oncostatin M (OSM) (Peprotech) plus 10^−6^ M Dexamethasone (DEX, Sigma-Aldrich) treatment for another 5 days. The differentiation media were changed every 2 days.

### siRNA transfection

hASCs were plated at 20,000 cells/cm^2^ in antibiotic-free basal medium 24 hours prior to transfection. siRNA transfection was performed following the manufacturer’s protocol as previously described^34^. Briefly, ON-TARGET SMARTpool siRNAs directed against *β-catenin* (L-003482-00-0005, Dharmacon, Lafayette, USA) or Non-targeting siRNAs (D-001810-10-05, Dharmacon) were mixed with Transfection DharmaFECT 1 (Dharmacon), respectively. After a 20 minutes incubation at room temperature, the complexes were added to the cells at a final siRNA concentration of 25 nM. The medium was replenished with medium containing antibiotic 24 hours post-transfection. The culture medium was changed every 2 days for the duration of the experiment.

### Immunofluorescence

The cells were fixed with 4% paraformaldehyde for 20 minutes at room temperature, followed by permeabilization with 0.3% Triton X-100 in PBS for 5 minutes. The cells were rinsed and blocked with 10% goat serum (Zsgb-Bio, Beijing, China), 1% gelatin (Sigma-Aldrich) or 5% BSA for 60 minutes at room temperature separately. The cells were then incubated with rabbit anti-β-catenin (Cell Signalling Technology, Danvers, MA,USA) at 1:100, rabbit anti-GATA4 (Merck Millipore, Germany) at 1:100, rabbit anti-FOXA2 (Abgent, San Diego, CA, USA) at 1:50, mouse anti-HNF4α (Santa Cruz Biotechnology, Santa Cruz, CA, USA) at 1:50, rabbit anti-AFP (Dako, Glostrup, Denmark) at 1:200, rabbit anti-ALB (Sigma-Aldrich) at 1:200, rabbit anti-GSTA2 (Abgent) at 1:50, rabbit anti-CYP2B6 (Santa Cruz) at 1:50, mouse anti-CYP2A6 (Origene, Maryland, USA) at 1:50, goat anti-CYP2C9/19 (Santa Cruz) at 1:50, and goat anti-CYP3A4 (Santa Cruz) at 1:50 at 4 °C overnight. Following three 5-minutes washes in PBS with gentle agitation, an Alexa Fluor-conjugated secondary antibody (Invitrogen) at 1:500 was added, and the samples were incubated for 1 hour at 37 °C. The nuclei were counter-stained with DAPI (Sigma-Aldrich). The stained cells were examined under a Leica TCS SP8 confocal microscope (Leica, Wetzlar, Germany).

Quantitative analysis of immunofluorescence was performed by a Cellomics^®^ Arrayscan VTI HCS Reader using the standard acquisition camera mode (20× objective) (Thermo Scientific, USA). The quantitative features of anti-β-catenin and anti-GATA4 staining were assessed using Cellomics^®^ Morphology Explorer (Thermo). Data showed a relative florescent density of protein in cell or in nucleus from 10 random fields (n = 200 cells in each group) from three replicate wells in three independent experiments.

### Flow cytometry

For flow cytometric detection of CXCR4 (BD Biosciences, San Jose, CA) and AFP (Dako), the cells were treated as previously described^10^. For flow cytometric detection of transcription factors FOXA2 and HNF4α, the cells (1 × 10^6^ cells) were fixed using fixation/permeabilization working solution (Affymetrix eBioscience, Inc., San Diego, CA, USA) for 60 minutes. After being washed twice with permeabilization working solution, cells were blocked with 2% normal goat serum for 60 minutes at room temperature, then adding mouse anti-HNF4α (Santa Cruz) at 1:50, rabbit anti-FOXA2 (Abgent) at 1:50, or isotype control antibody (BD) separately, and incubated with at room temperature for 60 minutes. Following two 5-minutes washes in permeabilization working solution (Affymetrix eBioscience) with gentle agitation, an Alexa Fluor-conjugated secondary antibody (Invitrogen) at 1:500 was added, and the samples were incubated for 60 minutes at room temperature. Following two 5-minutes washes in permeabilization working solution. The cell suspensions were resuspended in permeabilization working solution for flow cytometry (BD Accuri C6, BD Biosciences) using FLOWJO^TM^ software (TreeStar Inc., Ashland, USA).

### Real-time RT-PCR

Real-time RT-PCR was performed as previously described^10^,^34^. Total cellular RNA was extracted from 2.5 × 10^5^ cells with the RNeasy Mini Kit (QIAGEN, Hilden, Germany) according to the manufacturer’s instructions. For PCR analysis, 1 μg of RNA was reverse-transcribed to cDNA using Superscript III reverse transcriptase and random hexamer primers (Invitrogen). Real-time PCR analysis was performed on an ABI Prism 7300 Sequence Detection System using the SYBR Green PCR Master Mix (Applied Biosystems, Foster City, CA). The reaction consisted of 10 μL of SYBR Green PCR Master Mix, 1 μL of a 5 μM mix of forward and reverse primers, 8 μL water, and 1 μL template cDNA in a total volume of 20 μL. Cycling was performed using the default conditions of ABI 7300 SDS Software 1.3.1. The relative expression of each gene was normalized against 18 S rRNA. The data are presented as the mean ± s.d. The primers used are listed in Supplemental Table S1.

### LDL uptake cell-based assay

For LDL uptake cell-based assay, the cells were treated with LDL-DyLight^TM^ 550 (Cayman chemical, Ann Arbor, Michigan, USA) working solution in serum-free medium. Incubate the cells at 37 °C with 5% CO_2_ for an additional 18 hours. At the end of LDL uptake incubation, the culture medium was replaced with fresh culture medium. The degree of LDL uptake was examined under a Leica TCS SP8 confocal microscope (Leica, Wetzlar, Germany) with filters capable of measuring excitation and emission wavelengths 540 and 570 nm, respectively.

For LDL receptors examination, the cells were washed with TBS briefly, and fixed with cell-based assay fixative solution (Cayman chemical) for 10 minutes. Wash the cells with TBST three times for five minutes each. Incubate the cells for one hour with rabbit anti-LDL receptor antibody (Cayman chemical). Wash the cells with TBST three times for five minutes each. Incubate the cells for one hour with Dylight^TM^ 488-conjugated secondry antibody (Cayman chemical). Wash the cells with TBST three times for five minutes each. The nuclei were counter-stained with DAPI (Sigma-Aldrich). The stained cells were examined under a Leica TCS SP8 confocal microscope (Leica, Wetzlar, Germany).

### ALB analysis

Medium was harvested following 24 hours of culture of the different cell populations. The ALB content of the culture supernatants was quantified using a commercially available enzyme-linked immunosorbent assay kit (Alpha Diagnostic Intl, San Antonio, TX, USA) according to the manufacturer’s protocol.

### Glycogen synthesis

Glycogen synthesis was evaluated utilizing glycogen colorimetric/fluorometric assay kit (BioVision incorporated, Milpitas, CA, USA) according to the manufacturer’s instructions.

### CYP450 activity assay

CYP2C9 and CYP3A4 enzyme activity were assessed by P450-Glo assay (Promega, Madison, Wisconsin, USA), according to the manufacturer’s instructions. Differentiated cells were treated with rifampicin (25 μM, Sigma-Aldrich) for 72 hours and media were changed every day. The cells were incubated at 37 °C in fresh medium with Luciferin-H for 4 hours or Luciferin-IPA for 60 minutes separately. After the incubation, 50 μL of medium was transferred to a 96-well plate and mixed with 50 μL of luciferin detection reagent to initiate the luminescent reaction. After 60 minutes of incubation at 37 °C, the luminescence was measured with a luminometer (Envision2104-0010, Waltham, Massachusetts, USA).

### Statistical analysis

At least three independent determinations of each parameter were compared among the treatment groups by one-way ANOVA using the statistical software SPSS 11.5 (IBM Corporation, Armonk, NY, USA). Differences were considered significant if *p* < 0.05.

## Additional Information

**How to cite this article**: Huang, J. *et al*. Activation of Wnt/β-catenin signalling via GSK3 inhibitors direct differentiation of human adipose stem cells into functional hepatocytes. *Sci. Rep.* **7**, 40716; doi: 10.1038/srep40716 (2017).

**Publisher's note:** Springer Nature remains neutral with regard to jurisdictional claims in published maps and institutional affiliations.

## Supplementary Material

Supplementary Information

## References

[b1] Elaut G. . Molecular mechanisms underlying the dedifferentiation process of isolated hepatocytes and their cultures. Curr Drug Metab 7, 629–660, doi: 10.2174/138920006778017759 (2006). 16918317

[b2] Banas A. . Adipose tissue-derived mesenchymal stem cells as a source of human hepatocytes. Hepatology 46, 219–228, doi: 10.1002/hep.21704 (2007). 17596885

[b3] D’Souza N. . Mesenchymal stem/stromal cells as a delivery platform in cell and gene therapies. BMC Med 13, 186, doi: 10.1186/s12916-015-0426-0 (2015). 26265166 PMC4534031

[b4] Clynes M. Cell culture models for study of differentiated adipose cells. Stem Cell Res Ther 5, 137, doi: 10.1186/scrt527 (2014). 25809002 PMC4396124

[b5] Xu D. . Enabling autologous human liver regeneration with differentiated adipocyte stem cells. Cell Transplant 23, 1573–1584, doi: 10.3727/096368913X673432 (2014). 24148223

[b6] Gordillo M., Evans T. & Gouon-Evans V. Orchestrating liver development. Development 142, 2094–2108, doi: 10.1242/dev.114215 (2015). 26081571 PMC4483763

[b7] Holtzinger A. . New markers for tracking endoderm induction and hepatocyte differentiation from human pluripotent stem cells. Development 142, 4253–4265, doi: 10.1242/dev.121020 (2015). 26493401 PMC4689216

[b8] Zorn. Liver development in StemBook (ed. The Stem Cell Research Community, StemBook) pp. doi/10.3824/stembook.1.25.1. (The Stem Cell Research Community, 2008) Available at: http://www.stembook.org/node/512. (Accessed: 31th October 2008).

[b9] Hoepfner J. . Biphasic modulation of Wnt signaling supports efficient foregut endoderm formation from human pluripotent stem cells. Cell Biol Int 40, 534–548, doi: 10.1002/cbin.10590 (2016). 26861571

[b10] Li X. . Direct differentiation of homogeneous human adipose stem cells into functional hepatocytes by mimicking liver embryogenesis. J Cell Physiol 229, 801–812, doi: 10.1002/jcp.24501 (2014). 24166453

[b11] Bone H. K., Nelson A. S., Goldring C. E., Tosh D. & M. J. Welham A novel chemically directed route for the generation of definitive endoderm from human embryonic stem cells based on inhibition of GSK-3. J Cell Sci 124, 1992–2000, doi: 10.1242/jcs (2011). 21610099 PMC3104033

[b12] Siller R., Greenhough S., Naumovska E. & Sullivan G. J. Small-molecule-driven hepatocyte differentiation of human pluripotent stem cells. Stem cell reports 4, 939–952, doi: 10.1016/j.stemcr.2015.04.001 (2015). 25937370 PMC4437467

[b13] Lian X. . Efficient differentiation of human pluripotent stem cells to endothelial progenitors via small-molecule activation of WNT signaling. Stem cell reports 3, 804–816, doi: 10.1016/j.stemcr.2014.09.005 (2014). 25418725 PMC4235141

[b14] Kunisada Y., Tsubooka-Yamazoe N., Shoji M. & Hosoya M. Small molecules induce efficient differentiation into insulin-producing cells from human induced pluripotent stem cells. Stem Cell Res 8, 274–284, doi: 10.1016/j.scr.2011.10.002 (2012). 22056147

[b15] Hay D. C. . Highly efficient differentiation of hESCs to functional hepatic endoderm requires ActivinA and Wnt3a signaling. Proc Natl Acad Sci USA 105, 12301–12306, doi: 10.1073/pnas.0806522105 (2008). 18719101 PMC2518825

[b16] Baxter M. . Phenotypic and functional analyses show stem cell-derived hepatocyte-like cells better mimic fetal rather than adult hepatocytes. J Hepatol 62, 581–589, doi: 10.1016/j.jhep.2014.10.016 (2015). 25457200 PMC4334496

[b17] Touboul T. . Stage-specific regulation of the WNT/beta-catenin pathway enhances differentiation of hESCs into hepatocytes. J Hepatol 64, 1315–1326, doi: 10.1016/j.jhep.2016.02.028 (2016). 26921690 PMC5010388

[b18] McCubrey J. A. . Multifaceted roles of GSK-3 and Wnt/beta-catenin in hematopoiesis and leukemogenesis: opportunities for therapeutic intervention. Leukemia 28, 15–33, doi: 10.1038/leu.2013.184 (2014). 23778311 PMC3887408

[b19] So J., Martin B. L., Kimelman D. & Shin D. Wnt/beta-catenin signaling cell-autonomously converts non-hepatic endodermal cells to a liver fate. Biol Open 2, 30–36, doi: 10.1242/bio.20122857 (2013). 23336074 PMC3545266

[b20] Lade A. G. & Monga S. P. Beta-catenin signaling in hepatic development and progenitors: which way does the WNT blow? Dev Dyn 240, 486–500, doi: 10.1002/dvdy.22522 (2011). 21337461 PMC4444432

[b21] Thompson M. D. & Monga S. P. WNT/beta-catenin signaling in liver health and disease. Hepatology 45, 1298–1305, doi: 10.1002/hep.21651 (2007). 17464972

[b22] Wu D. & Pan W. GSK3: a multifaceted kinase in Wnt signaling. Trends Biochem Sci 35, 161–168, doi: 10.1016/j.tibs.2009.10.002 (2010). 19884009 PMC2834833

[b23] Morrison G., Scognamiglio R., Trumpp A. & Smith A. Convergence of cMyc and beta-catenin on Tcf7l1 enables endoderm specification. EMBO J 35, 356–368, doi: 10.15252/embj.201592116 (2016). 26675138 PMC4741304

[b24] Yu C., Liu K., Tang S. & Ding S. Chemical approaches to cell reprogramming. Curr Opin Genet Dev 28, 50–56, doi: 10.1016/j.gde.2014.09.006 (2014). 25461450 PMC4747244

[b25] Snykers S., De Kock J., Rogiers V. & Vanhaecke T. *In vitro* differentiation of embryonic and adult stem cells into hepatocytes: state of the art. Stem Cells 27, 577–605, doi: 10.1634/stemcells.2008-0963 (2009). 19056906 PMC2729674

[b26] D’Amour K. A. . Efficient differentiation of human embryonic stem cells to definitive endoderm. Nat Biotechnol 23, 1534–1541, doi: 10.1038/nbt1163 (2005). 16258519

[b27] Li W., Li K., Wei W. & Ding S. Chemical approaches to stem cell biology and therapeutics. Cell stem cell 13, 270–283, doi: 10.1016/j.stem.2013.08.002 (2013). 24012368 PMC3898630

[b28] Huang T. S. . A Regulatory Network Involving beta-Catenin, e-Cadherin, PI3k/Akt, and Slug Balances Self-Renewal and Differentiation of Human Pluripotent Stem Cells In Response to Wnt Signaling. Stem cells 33, 1419–1433, doi: 10.1002/stem.1944 (2015). 25538040 PMC5297972

[b29] Ling L., Nurcombe V. & Cool S. M. Wnt signaling controls the fate of mesenchymal stem cells. Gene 433, 1–7, doi: 10.1016/j.gene.2008.12.008 (2009). 19135507

[b30] Levinson-Dushnik M. & Benvenisty N. Involvement of hepatocyte nuclear factor 3 in endoderm differentiation of embryonic stem cells. Mol Cell Biol 17, 3817–3822 (1997). 9199315 10.1128/mcb.17.7.3817 PMC232233

[b31] Xuan S. & Sussel L. GATA4 and GATA6 regulate pancreatic endoderm identity through inhibition of hedgehog signaling. Development 143, 780–786, doi: 10.1242/dev.127217 (2016). 26932670 PMC4813334

[b32] Morisco C. . Glycogen synthase kinase 3beta regulates GATA4 in cardiac myocytes. J Biol Chem 276, 28586–28597, doi: 10.1074/jbc.M103166200 (2001). 11382772

[b33] Nelson L. J. . Acetaminophen cytotoxicity is ameliorated in a human liver organotypic co-culture model. Sci Rep 5, 17455, doi: 10.1038/srep17455 (2015). 26632255 PMC4668374

[b34] Hua M. . Molecular mechanisms regulating the establishment of hepatocyte polarity during human hepatic progenitor cell differentiation into a functional hepatocyte-like phenotype. J Cell Sci 125, 5800–5810, doi: 10.1242/jcs.110551 (2012). 22976305

