# Supplementary material for: Activation of Wnt/β-catenin signalling via GSK3 inhibitors direct differentiation of human adipose stem cells into functional hepatocytes
Source: Sci Rep. 2017 Jan 17;7:40716. doi: 10.1038/srep40716 (PMC5240561; doi:10.1038/srep40716)
Supplement: Supplementary Information [file srep40716-s1.doc]

**Activation of Wnt/β-catenin signalling via GSK3 inhibitors direct differentiation of human adipose stem cells into functional hepatocytes**

Jieqiong Huang^1^, Xinyue Guo^1^, Weihong Li, Haiyan Zhang*

**Supplementary information**

**
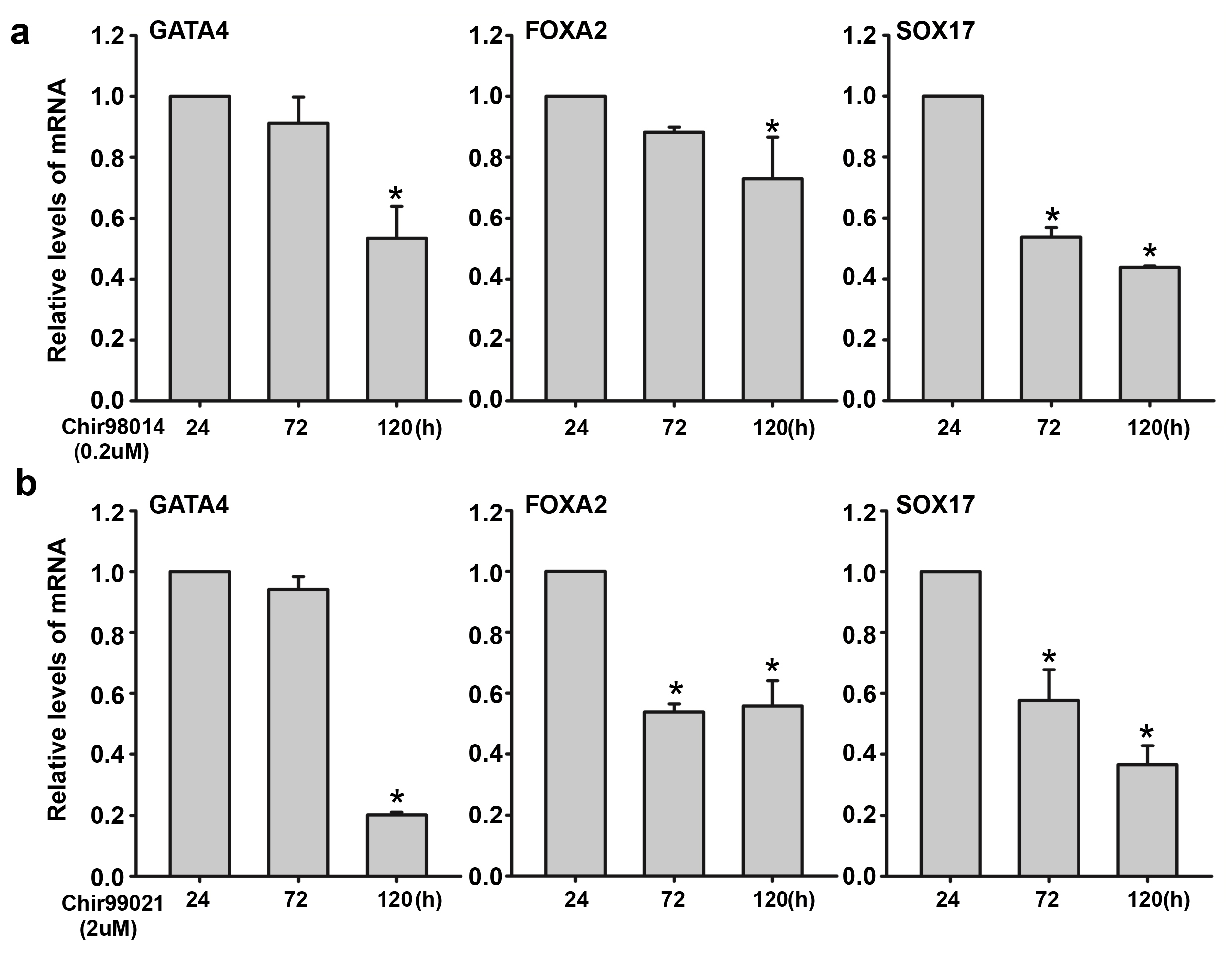
**

**Figure S1. Temporal activation of Wnt/β-catenin signalling is conducive to hASCs differentiation.** Real-time RT-PCR analysis of the GATA4, FOXA2 and SOX17 mRNA levels in hASCs after treatment with Chir98014 (a) or Chir99021 (b) for different times. The relative expression of each gene was normalized to 18S. *, Statistical significance compared to the cells after treatment for 24 hours, *p*<0.05.


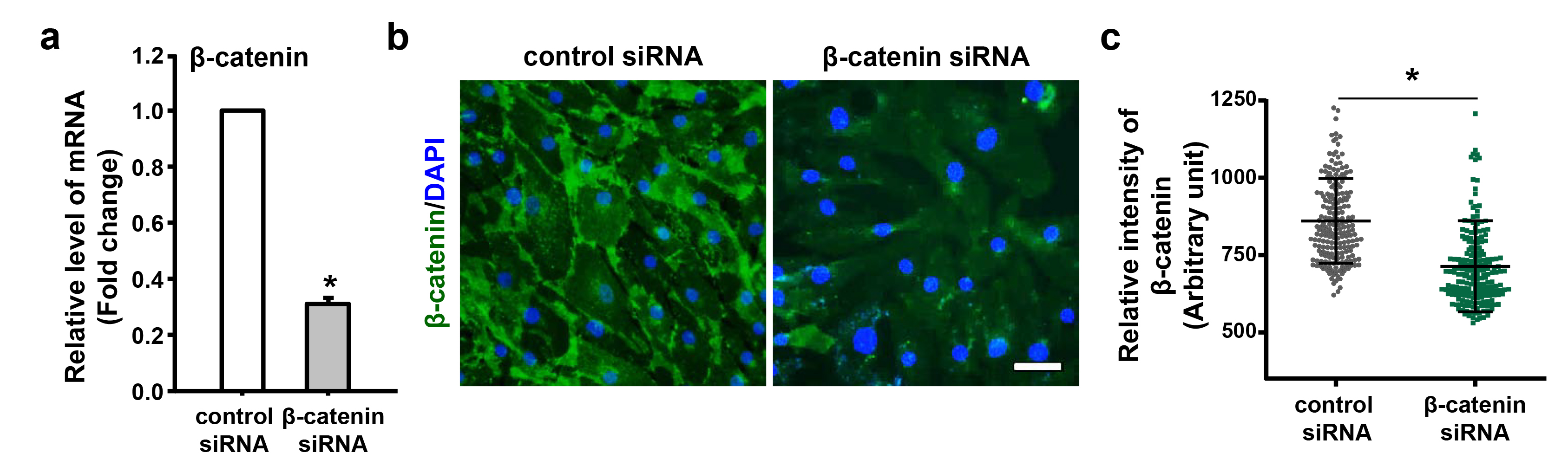


**Figure S2. Efficiency of β-catenin siRNA transfection in hASCs.** (a) Real-time RT-PCR analysis of the β-catenin mRNA levels in hASCs 24 hours post-siRNA transfection. The relative expression of each gene was normalized to 18S. *, Statistical significance compared to the control siRNA, *p*<0.05. (b) Immunofluorescence staining for β-catenin was examined 72 hours post-siRNA transfection in β-catenin siRNA-treated cells and control siRNAs treated cells. Scale bars, 100 μm. (c) Quantitative analysis of β-catenin was determined using the HCS platform 72 hours post-siRNA transfection in β-catenin siRNA-treated cells and control siRNAs-treated cells. Data showed a relative florescent density of β-catenin. n=200 cells in each group. *, Statistical significance compared to the control siRNA, *p*<0.05.


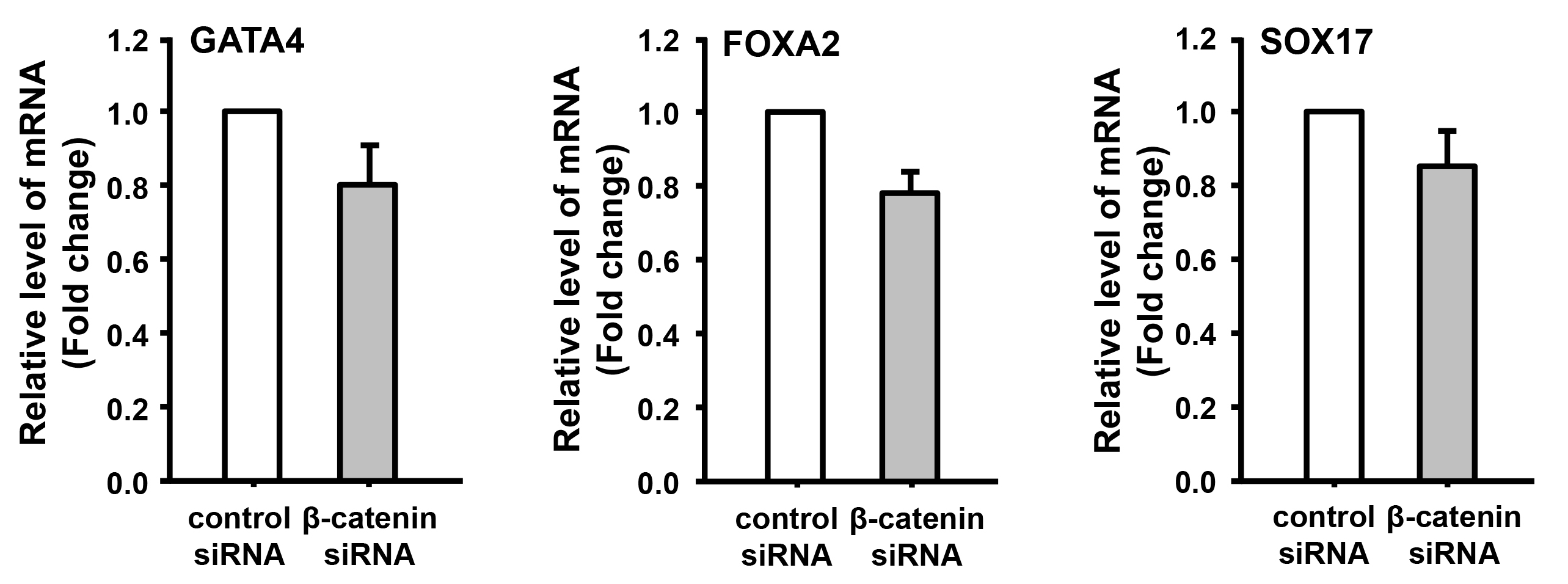


**Figure S3. The effect of β-catenin siRNA on the expression of definitive endoderm specific genes.** Real-time RT-PCR analysis of the mRNA levels of definitive endoderm specific genes in hASCs 24 hours post-siRNA transfection. The relative expression of each gene was normalized to 18S.

**
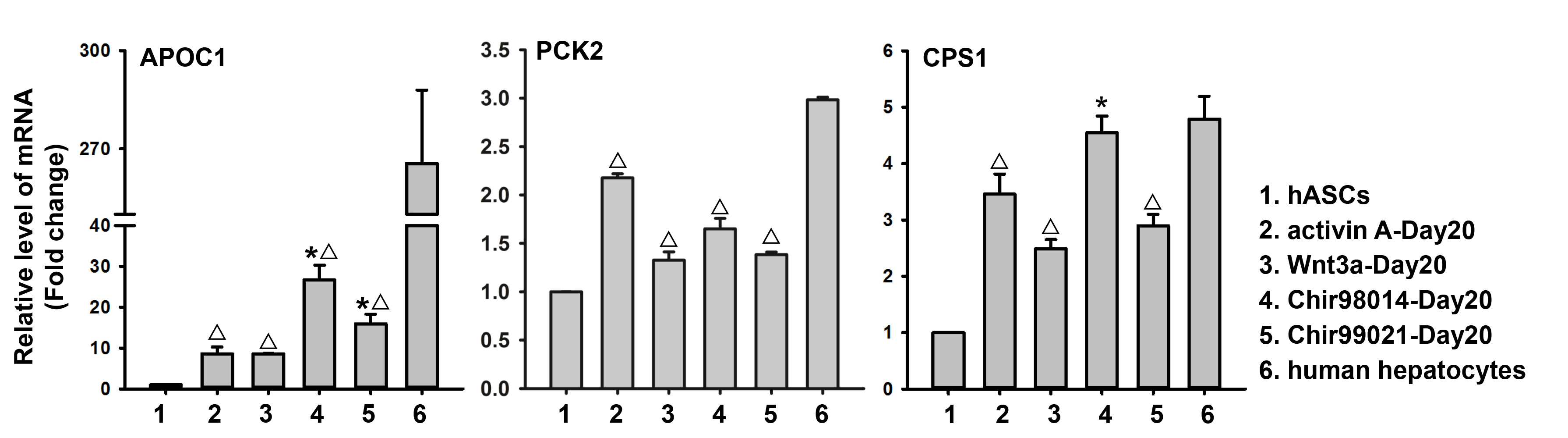
**

**Figure S4. Characterization of hASC-HLCs.** Real-time RT-PCR analysis of the APOC1, PCK2, and CPS1 mRNA level in hASCs, differentiated cells at day 20, and human hepatocytes. The relative expression of each gene was normalized to 18S. *, Statistical significance compared to hASCs, *p*<0.05; ^Δ^, Statistical significance compared to human hepatocytes, *p*<0.05.


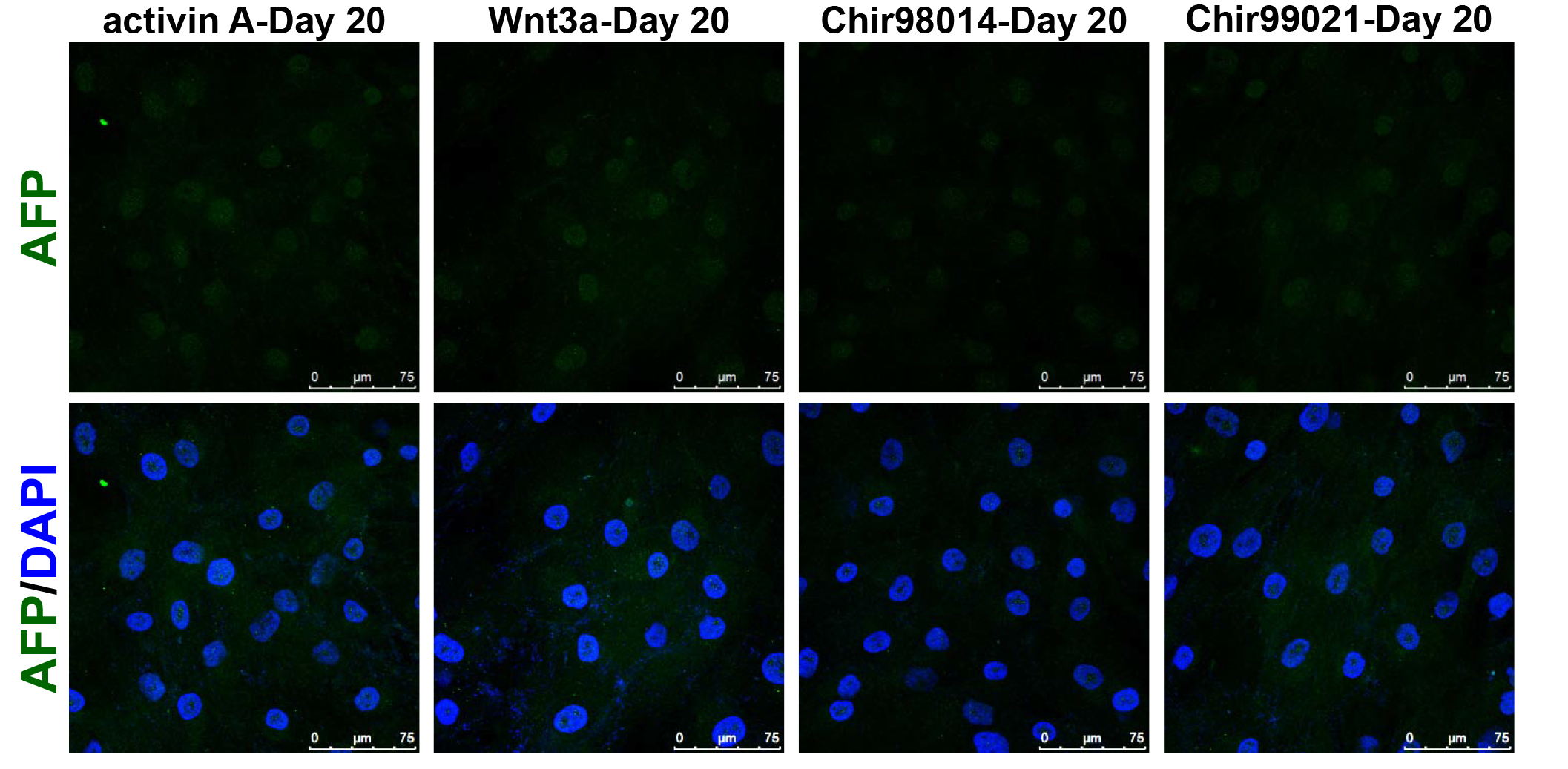


**Figure S5.** **Expression of AFP in differentiated cells.** Immunofluorescence staining for AFP in differentiated cells at day 20 with different factors. Bars, 75 μm.

**
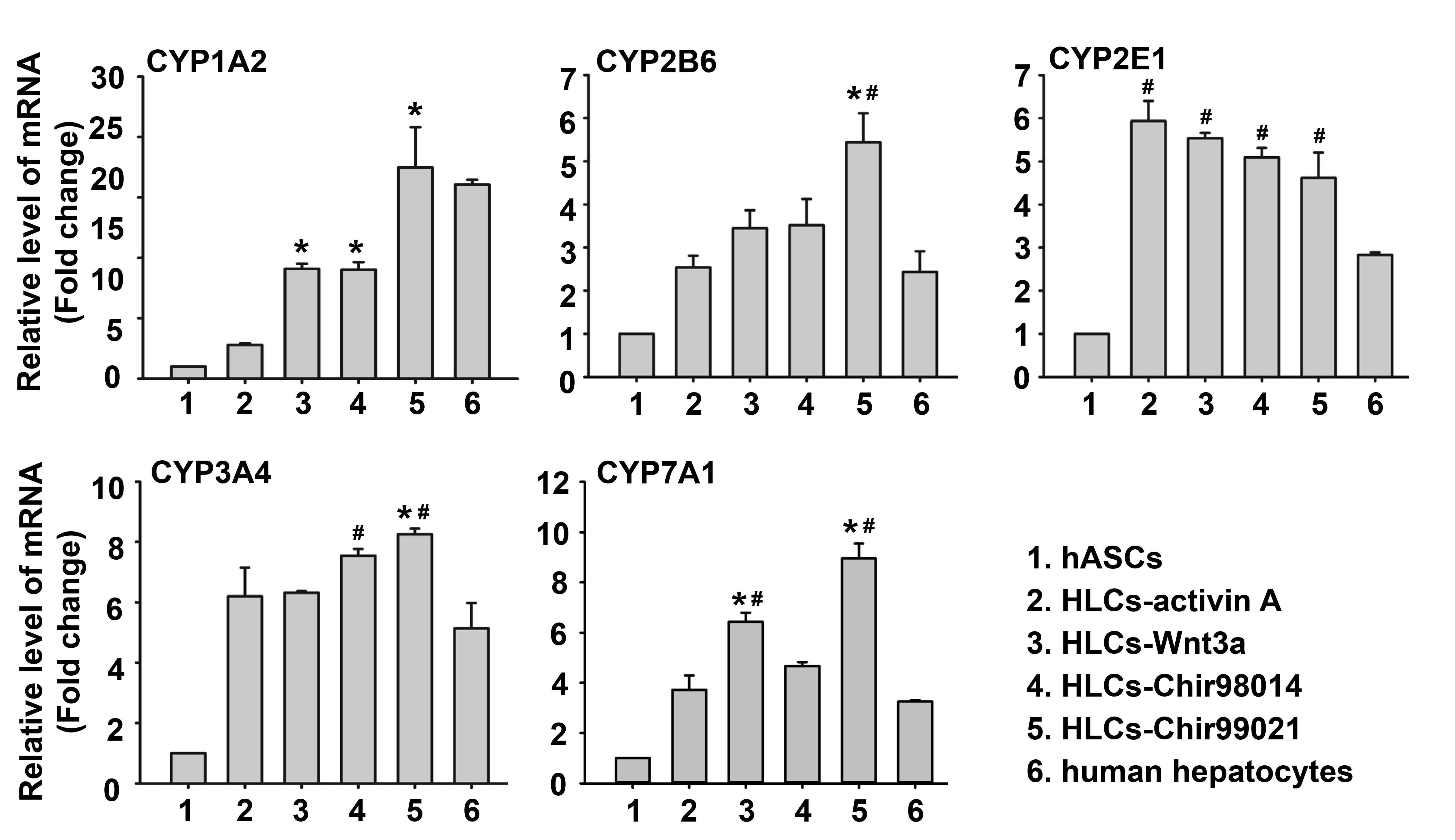
**

**Figure S6.** **Expression of CYP450 in hASC-HLCs.** Real-time RT-PCR analysis of the levels of CYP1A2, CYP2B6, CYP2E1, CYP3A4, CYP3A7, and CYP7A1 mRNA in in hASCs, hASC-HLCs, and human hepatocytes. The relative expression of each gene was normalized against 18S. *, Statistical significance compared to hASC-HLCs-activin A,*p*<0.05. ^#^, Statistical significance compared to human hepatocyte,*p*<0.05.

**Table S1. Primers for Real-Time RT-PCR**

| Accession number | Name | 5’-Sequence-3’ |
| --- | --- | --- |
| NM_001645.3 | APOC1 | F:GGTCCTGGTGGTGGTTCT  R:TGTTTGATGCGGCTGATG |
| NM_004655.3 | AXIN2 | F:TGACGGACAGCAGTGTAGATG  R:TTCTCGGGAAATGAGGTAGAG |
| NM_001875.4 | CPS1 | F:CTGACCCTGCCTACAAAG  R:CACCAGCAAACCTGAAAC |
| NM_001012329.1 | CTNNB | F:ATTGTCCACGCTGGATTTTC  R:TCGAGGACGGTCGGACT |
| NM_001008540 | CXCR4 | F: AGGCTGCACCTGTCAGTGGCCGA  R:GCGTGGACGATGGCCAGGTAGCG |
| NM_000761.3 | CYP1A2 | F:AGTCTGTTCCCTTCTCGG  R: GGCTCTGGTGGACTTTT |
| NM_000767 | CYP2B6 | F: AGGGAGATTGAACAGGTGATT  R:GATTGAAGGCGTCTGGTTT |
| NM_000773 | CYP2E1 | F: CTGACCACCCTCCGGAACTAT  R: GGCCTTGGGTCTTCCTGAGT |
| NM_001202855.2 | CYP3A4 | F:GGCGGATGTTGAAGTGAG  R:GTTGGGTGTTGAGGATGG |
| NM_000780 | CYP7A1 | F:GAGAAGGCAAACGGGTGAAC  R:GCACAACACCTTATGGTATGACA |
| NM_021784 | FOXA2 | F:CGCCCACTTCCAACTACCGC  R:GGCTCGTGCCCTTCCATCTT |
| NM_002052.3 | GATA4 | F:CCACAAGGCTATGCGTCTC  R:CTTCTTTGCTATCCTCCAAGTC |
| NM_016269.4 | LEF1 | F:TGGCATCCCTCATCCAGCTATTGT  R:TGAGGCTTCACGTGCATTAGGTCA |
| NM_001018073.1 | PCK2 | F:TTCCCCACCGCACATACC  R:CCACCACCAATCCCAACG |
| NM_022454.3 | SOX17 | F:CAAGGGCGAGTCCCGTAT  R:CGACTTGCCCAGCATCTT |
| NM_003202 | TCF7 | F:AGAGAAGGAGGCTAAGAAGCCAGT R:ACTCAGCAATGACCTTGGCTCTCA |
|  | 18S | F:GTAACCCGTTGAACCCCATT  R:CCATCCAATCGGTAGTAGCG |
